# Molecular Typing, Antibiogram and PCR-RFLP Based Detection of *Aeromonas hydrophila* Complex Isolated from *Oreochromis niloticus*

**DOI:** 10.3390/pathogens9030238

**Published:** 2020-03-22

**Authors:** Abdelazeem M. Algammal, Mohamed Fathi Mohamed, Basma A. Tawfiek, Wael N. Hozzein, Waleed M. El Kazzaz, Mahmoud Mabrok

**Affiliations:** 1 Department of Bacteriology, Immunology and Mycology, Faculty of Veterinary Medicine, Suez Canal University, Ismailia 41522, Egypt; 2 National Institute of Oceanography and Fisheries, Suez 43511, Egypt; aquavet@hotmail.com (M.F.M.); basmatawfiek444@yahoo.com (B.A.T.); 3 Bioproducts Research Chair, Zoology Department, College of Science, King Saud University, Riyadh 11451, Saudi Arabia; hozzein29@yahoo.com; 4 Botany and Microbiology Department, Faculty of Science, Beni-Suef University, Beni-Suef 62511, Egypt; 5 Botany Department, Faculty of Science, Suez Canal University, Ismailia 41522, Egypt; walid_elkazaz@sience.suez.edu.eg; 6 Department of Fish Diseases and Management, Faculty of Veterinary Medicine Suez Canal University, Ismailia 41522, Egypt; dr.mahmoudmabrok@yahoo.com; 7 Fish Infectious Diseases Research Unit (FID RU), Faculty of Veterinary Science, Chulalongkorn University, Bangkok 10330, Thailand

**Keywords:** *A. hydrophila* complex, *O. niloticus*, PCR, RFLP, antibiogram

## Abstract

Motile Aeromonas septicemia is a common bacterial disease that affects *Oreochromis niloticus* and causes tremendous economic losses globally. In order to investigate the prevalence, molecular typing, antibiogram and the biodiversity of *Aeromonas hydrophila* complex, a total of 250 tilapia (*Oreochromis niloticus*) were collected randomly from 10 private tilapia farms (25 fish/farm) at El-Sharkia Governorate, Egypt. The collected fish were subjected to clinical and bacteriological examinations. The majority of infected fish displayed ulcerative necrosis, exophthalmia, and internal signs of hemorrhagic septicemia. The prevalence of *A. hydrophia* complex was 13.2%, where the liver was the most predominant affected organ (54.1%). Polymerase chain reaction (PCR) was used to verify the identification of *A. hydrophila* complex using one set of primers targeting *gyr*B as well as the detection of virulent genes (*aer*A, *alt*, and *ahp*). All isolates were positive for the *gyr*B-conserved gene and harbored *aer*A and *alt* virulence genes. However, none of those isolates were positive for the *ahp* gene. The antimicrobial sensitivity was carried out, where the recovered strains were completely sensitive to ciprofloxacin and highly resistant to amoxicillin. All retrieved strains showed the same phenotypic characteristics and were identical based on the restriction fragment length polymorphism (RFLP). Experimentally challenged fish presented a high mortality rate (76.67%) and showed typical signs as in naturally infected ones. In conclusion, the synergism of phenotypic and genotypic characterization is a valuable epidemiological tool for the diagnosis of *A. hydrophila* complex. RFLP is a fundamental tool for monitoring the biodiversity among all retrieved strains of *A. hydrophia*.

## 1. Introduction

Aquaculture is one of the most significant food-delivering resources that could outfit the total populace with a protein of animal origin and decrease food deficiency due to overpopulation. Actually, about 16% of the animal protein consumed globally is derived from fish, and more than a billion persons depend on fish as the main source of protein [1]. Tilapia is the second most cultivated fish in aquaculture after cyprinid and its production has quadrupled over the past decade because of its sustainability, economical price, palatability, and the ease of cultivation [2]. In Egypt, tilapia is the main component of pisciculture, whether in fresh or brackish water resources, farms, and fish cages [3]. Like other organisms, tilapia is spontaneously exposed to various diseases, especially induced by the presence of bacteria [4,5]. *Aeromonas hydrophila*, the causative agent of Motile Aeromonas Septicemia is one of the most threatening pathogens that has caused great annual losses among fish populations [6]. The bacteria affect various fish species; including carp [7], catfish [8], tilapia [9], eels [10], and goldfish [11]. The most prominent signs associated with the disease are swelling of tissues, ascites, red sores, ulcerative necrosis, and hemorrhagic septicemia [12]. Identification of *Aeromonas* to the species level using conventional methods, showed great obstacles due to its phenotypic complexity and interspecies homogeneity [13]. Most of the commercially available identification systems can identify *Aeromonas* isolates to the level of *A. hydrophila* group or *A. hydrophila*/*A. caviae*. However, the results of these systems are usually miserable or erroneous due to scanty preferential markers to detect differences between species [14]. Additionally, single-use of API system (Biomerieux) can’t definitively identify *Aeromonas* species except if combined with several other tests [15]. Thus, molecular approaches including PCR and DNA sequencing and analysis have been developed for the precise diagnosis of *Aeromonas* infections [16]. 

Using such molecular techniques has led to a more refined identification and limiting all discrepancies associated with the biochemical identification of this organism [17]. Polymerase chain reaction (PCR) is a fundamental modern technique that has the ability to amplify the selected amplicons in order to confirm the diagnosis and/or the virulence of certain bacterial pathogens [18,19,20]. The most common selected genes are 16S rRNA and housekeeping genes. The genetic typing of *Aeromonas hydrophila* or *Aeromonas* sp using sets of primers targeting *GCAT*, 16S rRNA and *rpo*D genes were reported by [21]. Indeed, exploiting variations in homologous DNA sequences of any bacteria is a dynamic marker for tracking the transmission of the disease induced by these bacteria. Restriction fragment length polymorphism (RFLP) analysis is an extremely salutary and simplified technique that distinguishes between the most relevant and antigenically related organisms including bacteria [22]. The technique was recurrently used for genomic mapping, localization of genetic disorders, and evaluating the risk of such disease [23]. 

Although several studies briefly elucidate the incidence of *A. hydrophila* among different species, to the best of our knowledge, there are few studies concerned with the pathogenicity, molecular typing and biodiversity of *A. hydrophila* infection in *O. niloticus,* particularly in Egypt. The current study aimed to emphasize the prevalence, pathogenicity as well as the antibiogram of *A. hydrophila* complex in cultured *O. niloticus*. Likewise, the genetic typing of all retrieved strains and their intraspecific heterogeneity were evaluated using PCR based RFLP technique.

## 2. Materials and Methods

### 2.1. Fish Sampling 

A total of 250 tilapia (*Oreochromis niloticus*) weighing 120 ± 5 g were randomly collected from 10 private tilapia farms (25 fish/farm) located in two major aquaculture areas in El-Sharkia Governorate, Egypt, from May to October 2018. The first area (Abbassa) is located in the south of El-Sharkia, while the second one (El-Hossania) is located in the north of the same province. Both live and moribund fish were transferred immediately in insulating iceboxes to the laboratory of Fish Diseases at the National Institute of Oceanography and Fisheries, Suez, Egypt, and were subjected to the clinical and bacteriological examinations.

### 2.2. Clinical Examination

Fish were examined for the presence of external and internal pathological lesions following the methods described elsewhere by [24].

### 2.3. Bacteriological Examination

Fresh specimens were aseptically handled and dissected immediately upon arrival for bacteriological analysis. Loopful samples of kidneys, livers, and spleens were inoculated onto Tryptic Soy Agar (TSA, Oxoid, Hampshire, UK), Brain Heart Infusion Agar (BHIA, Oxoid), and MacConky’s Agar (Oxiod), and incubated at 37 °C for 24 h [25]. Separate colonies were precisely selected and subcultured on Rimler Shotts (RS) medium, specific for Aeromonads at 37 °C for an additional 24 h [26]. The pure colonies were harvested and identified morphologically (Gram’s staining) and biochemically using different sets of biochemical reactions, including cytochrome oxidase (Biomerieux, Marcy-l'Étoile, France), catalase, glucose fermentation, indole, methyl red, nitrate reduction, esculin hydrolysis, inositol fermentation, and the Voges Proskauer test. The sensitivity of different retrieved isolates to the various concentrations of sodium chloride (0–5%), vibriostatic agent (150 µg) and novobiocin was also considered. Additionally, the hemolytic activity of such a colony was investigated using TSA supplemented with 5% sheep red blood cells, whereas the active motility was examined following streaking on a slant semisolid [27].

### 2.4. Molecular Typing of A. hydrophila Complex and its Virulence Genes 

Genomic DNA of purified bacterial cells was extracted using the QIAamp DNA Mini Kit (Invitrogen, USA). Recovered DNA templates were quantified using a Nanodrop (Nanodrop 1000, Thermo Scientific, Loughborough, UK) adjusted to 100 ng μL^−1^ and kept frozen at −20 °C until being used for PCR. To verify that the recovered isolates belonged to *A. hydrophila,* one set of primers targeting the *gyr*B conserved gene was selected according to [28]. Moreover, the PCR detection of several virulent genes (*aer*A, *alt*, and *ahp*) associated with Aeromoniasis and related to its zoonotic probability was performed using different sets of primers described elsewhere by [29]. The oligonucleotides sequences of the primers and their PCR conditions are provided in Table 1. PCR reaction mixtures of 25 μL were amplified in the MJ Mini^TM^ Gradient Thermocycler apparatus (Biometra, Göttingen, Germany) using a commercial kit of Green Master Mix (NZYtech, Lisboa, Portugal). A reaction with DNA free template was used as a negative control, while a reference strain, kindly provided by the Animal Health Research Institute at Dokki, Cairo, Egypt was used as a positive control. Amplified fragments were screened by 1.5% (*w*/*v*) agarose gel electrophoresis (Applichem GmbH, Darmstadt, Germany) for 45 min at 100 V in 1× TAE buffer (0.04 M Tris, 0.0001 M EDTA, pH 8.0), visualized using 15 µL of DNA gel stain (Sigma-Aldrich) and photographed under UV transilluminator. A 100 bp ladder (Fermentas, Thermo Scientific, Darmstadt, Germany) was used.

### 2.5. Restriction Fragment Length Polymorphism (RFLP) of A. hyrophila Complex 

To exploit the variations in homologous DNA sequences and determine the degree of genetic diversity among 14 representative isolates of *A. hyrophila* (two isolates per each infected farm), the RFLP analysis of the amplified *gyr*B gene of those isolates was performed. Prior enzymes selections, the restriction cutting maps were analyzed using GenScript Restriction Enzyme Map Analysis Online Tools. Digestive enzyme acronym, sequence, site lengths, frequency, and cutting positions are provided in Table 2. The digestion of amplified products was performed using two restriction enzymes (EcoRII and Eco31I) following the manufacturer protocol of Thermo Scientific Company (Germany). Briefly, the PCR products were incubated with 2 μL of each digestive enzyme and 2 μL of the corresponding buffer supplied with the enzyme in a total volume of 20 μL at 37 °C for 60 min. To stop the reaction, the mixture was heat-inactivated at 65 °C for 20 min. The fragments patterns were screened by 2% (*w*/*v*) agarose gel electrophoresis (Applichem, GmbH, Germany,) for 1 h at 80 V in 1× TAE buffer (0.04 M Tris, 0.0001 M EDTA, pH 8.0). The data were interpreted using a gel documentation system (Photodoc-it, UVP, Cambridge, UK) and Total Lab Analysis software, www.totallab.com, (Ver.1.0.1).

### 2.6. Antimicrobial Susceptibility Testing

The sensitivity of all retrieved isolates to various commercial antimicrobial agents (Oxoid) including; ciprofloxacin, nalidixic acid, trimethoprim-sulphamethoxazole, erythromycin, amoxicillin, streptomycin, tetracycline, and chloramphenicol was performed in duplicates using a disc diffusion assay [30]. The reference strain *E. coli* ATCC 25922 was used as a control for the disc diffusion technique, which is kindly provided by Animal Health Research Institute, Egypt. The diameter of inhibition zone was measured by millimeter and expressed as sensitive, intermediate, and resistant as described by [31].

### 2.7. Pathogenicity Test

#### 2.7.1. Acclimation Period

A total of 120 apparently healthy *O. niloticus* weighing 115 ± 5 g with no history of a previous infection were collected from the World Fish center at Sharkia Governorate, Egypt. The fish were transferred to the National Institute of Oceanography and Fisheries, Suez Governorate, and left acclimated for two weeks prior to the challenge in a big fiberglass tank of 1000 L holding capacity. The tank was supplied with aerated, filtered, and dechlorinated tap water with an average salinity of 0.5 ± 0.12 g L^−1^. The water temperature was maintained at 25 ± 2 °C, while the dissolved oxygen (DO) was adopted at 5 ± 1 mg L^−1^ using automatic aerators (RINA, Genova, Italy). Water pH was adjusted at 7.2 and 12 h light/12 h dark cycle was adopted. Ammonia and nitrite levels were measured once a week using mercantile kits and never exceed 0.02 and 0.2 mg L^−1^, respectively. The fish were fed daily on commercial pellets (Skretting, Alexandria, Egypt) of 30% crude protein until visual satiety. The fecal matter and other debris were siphoned and 50% of the water was changed daily to minimize the ammonia toxicity. Fish that have normal reflexes and healthy appearance were selected for the challenge trial.

#### 2.7.2. Challenge Trial

The ethical approval was obtained from the National Institute of Oceanography and Fisheries, Suez. Ninety healthy Fish were distributed randomly into two identical groups in triplicate (45 fish/group), each contributed three-glass aquaria of 100 L and 15 fish holding capacity. The fish of the first group were injected intraperitoneally (IP) with 0.2 mL of sterile normal saline and served as a negative control, while the fish in the second group were injected IP with 0.2 mL of an overnight culture of *A. hydrophila* at a concentration of 3 × 10^8^ CFU mL^−1^ [32]. For inoculum preparation, bacteria with identical biochemical and molecular profiles to *A. hydrophila* were selected and were routinely cultured on tryptic soy broth (Oxoid) at 37 °C for 24 h. Subsequently, the bacterial suspension was adjusted to the final concentration using a 0.5 McFarland standard and by means of the Helber counting chamber. All fish groups were observed daily for 14 days post-challenge for the emergence of any pathological lesions and mortality. Moribund and newly dead fish were harvested, examined aseptically to verify the cause of death. Mortalities were considered only when the injected strain was recovered from infected fish (Koch’s postulates).

### 2.8. Statistical Analysis

The Chi-square was conducted to analyze the data to test the null hypothesis of different treatments using the statistical analysis software (SAS® software version 9.4). The significance level was (*P <* 0.05).

## 3. Results

### 3.1. Clinical Findings

**Figure 1:**
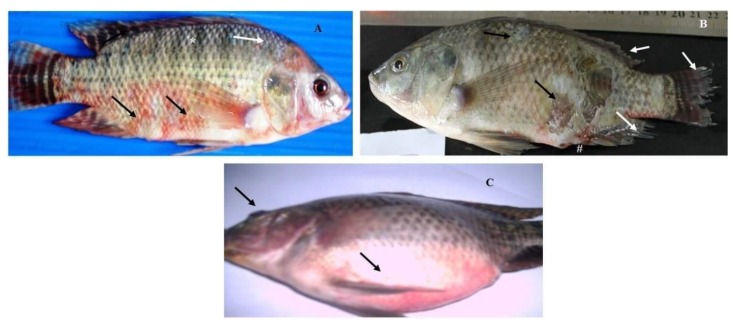
Naturally infected *O. niloticus* with *Aeromonas hydrophila* complex; (**A**) fish showing skin darkness (*), detached scales (white arrow), and external scattered hemorrhagic patches (black arrows); (**B**) fish showing ulcerative necrosis (black arrows), fin erosions (white arrows), and inflamed vent (#); (**C**) fish showing exophthalmia and abdominal distension (black arrows).

**Figure 2:**
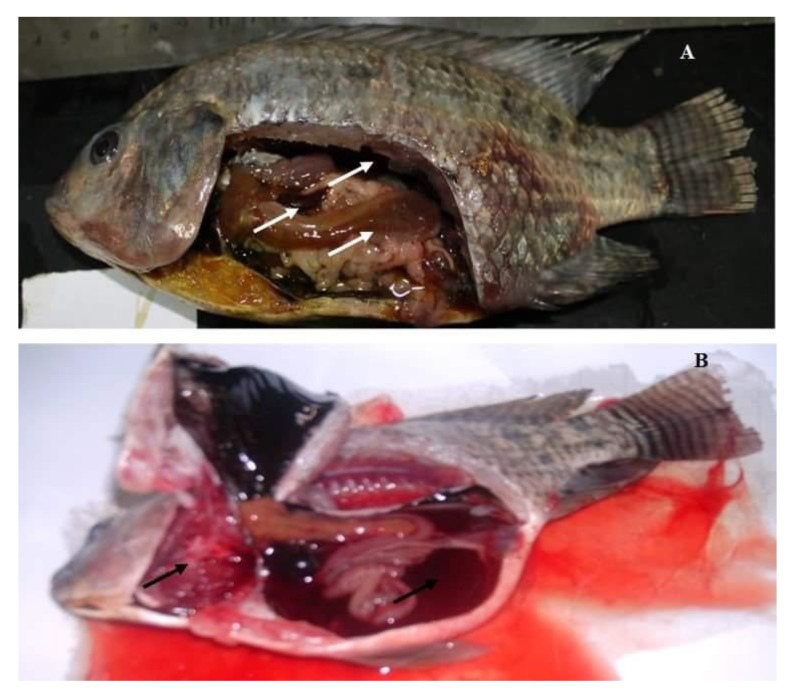
Naturally infected *O. niloticus* with *Aeromonas hydrophila* complex; (**A**) fish showing enlarged liver, engorged spleen, and congested kidney (white arrows); (**B**) fish showing sero-hemorrhagic fluids in the abdominal cavity and congested friable gills (Black arrows).

According to the progress of the disease, in acute infection, most of the naturally infected fish with *Aeromonas hydrophila* complex showed darkness of the skin, detachment of the scales, and large irregular hemorrhages on the external body surface (Figure 1A). In advanced stages, the hemorrhagic lesions were developed to shallow ulcers and many cases showed fin erosions, inflamed vent, exophthalmia, and abdominal distension (Figure 1B,C). The postmortem findings of naturally infected fish revealed enlarged liver, engorged spleen, and congested kidney (Figure 2A). Others showed sero-hemorrhagic fluids in the abdominal cavity and friable gills (Figure 2B).

### 3.2. Bacteriological Examination

All isolates were identified as *A. hydrophila* complex based on their morphology and biochemical characteristics. Microscopically, the bacteria appeared as Gram-negative, straight rod-shaped bacilli and Motile by single polar flagella. The bacteria grew well on RS medium and gave characteristic yellow circular colonies at 37 °C after 24 h. Additionally, all isolates showed *in vitro* high resistant patterns against vibriostatic agent (150 µg) and novobiocin. On blood agar, the colonies appeared as circular grayish in color due to beta-hemolysis and after a prolonged time of incubation, they turned to dark green. Biochemically as shown in Table 3, all isolates were positive with respect to catalase, cytochrome oxidase, aesculin hydrolysis, inositol, and Voges Proskauer, whereas they were negative to methyl red and H_2_S production. The bacteria reduced nitrates to nitrites without production of gas and showed high salt tolerance to the media supplemented with 0–4% (*w*/*v*) sodium chloride. Moreover, the bacteria had the ability to degrade casein, gelatin, and starch at 5–37 °C, and produced acid from fructose, sucrose, glucose, maltose, mannitol, salicin, and trehalose.

### 3.3. Prevalence of A. hydrophila Complex among Naturally Infected O. niloticus and Their Intensities in Different Internal Organs 

The results of the bacteriological analysis revealed that 33 out of 250 *O. niloticus* were positive to *A. hydrophila* complex with a total prevalence of 13.2%. The prevalence of bacteria and their patterns of distribution among different investigated farms were provided in Table 4. As indicated, 61 isolates of *A. hydrophila* complex were retrieved from seven infected farms, while only three farms were found free of infection. The statistical analysis proved that there is no significant difference in the prevalence of *A. hyrophila* between different farms at the first locality, while exhibiting a significant difference between various farms at the second locality (*P <* 0.05). In addition, there is no significant difference in the prevalence between the two localities (*P <* 0.05). The highest prevalence of bacteria was recorded in the liver (54.10%), followed by the kidney (26.30%), while the lowest prevalence was listed in the spleen. Statistically, there is a remarkable significant difference in the distribution of *A. hydrophila* in various internal organs (*P <* 0.0001) (Table 5).

### 3.4. Molecular Typing of A. hydrophila Complex and Their Virulent Genes

**Figure 3:**
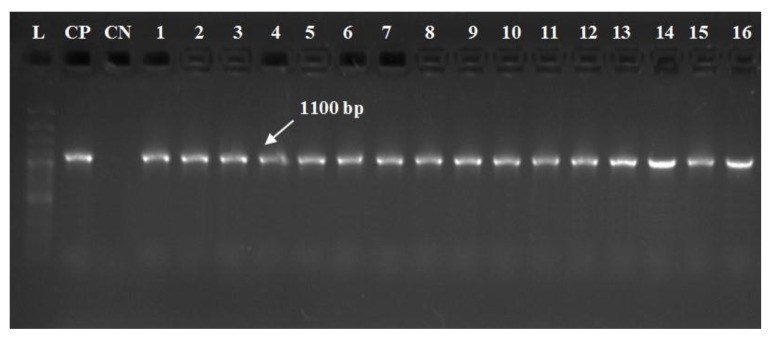
Electrophoretic pattern of *gyr*B gene of *A. hydrophila* complex using specific set of primer described elsewhere by Yanez et al. (2003). Lane marked L refer to 100 bp DNA ladder. CP is a control positive (*A. hydrophila* strain, kindly supplied by Animal Health Research Institute in Dokki, Cairo). CN is a negative control (DNA free template). Lanes 1–16, the specific DNA product amplified from the representative retrieved isolates of *O. niloticus* with expected amplicons size of 1100 bp.

**Figure 4:**
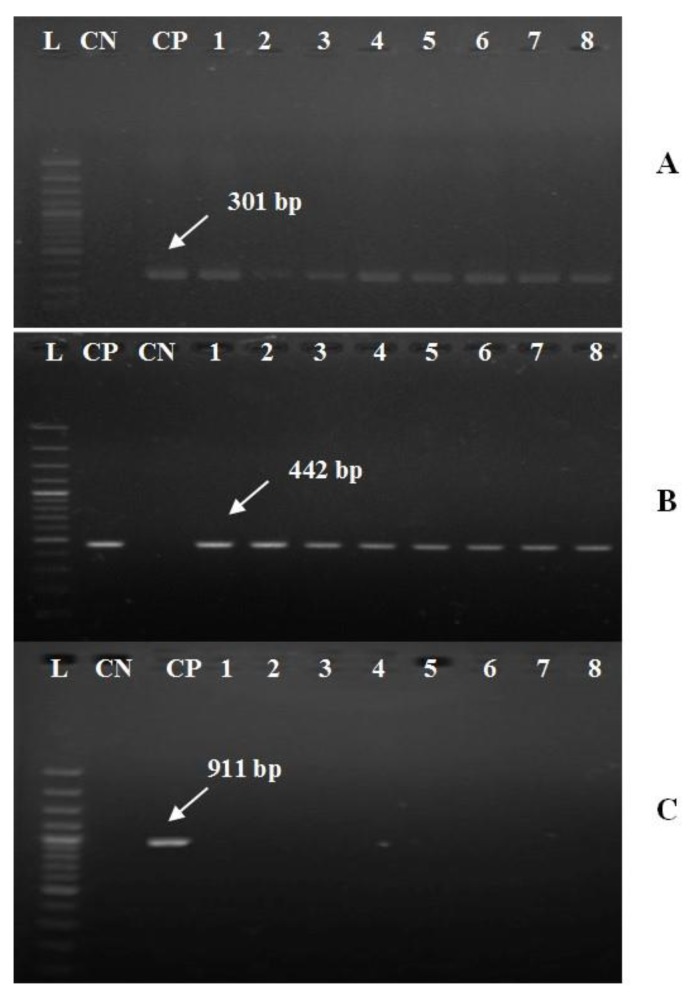
Electrophoretic pattern of *aer*A (**A**), *alt* (**B**), and *ahp* (**C**) virulent genes of *A. hydrophila* complex using specific sets of primers described elsewhere by Li, Ni, Liu and Lu [29]. Lanes marked L refer to 100 bp DNA ladder. CP is a control positive (*A. hydrophila* strain, kindly supplied by Animal Health Research Institute in Dokki, Cairo). CN is a negative control (DNA free template). Lanes 1–8, the specific DNA products amplified from the representative retrieved isolates of *O. niloticus* with expected amplicons size of 301, 442, and 911 bp, respectively.

All recovered strains were positive for *gyr*B, a conserved gene of *A. hydrophila* complex with a fragment size of 1100 bp (Figure 3). Additionally, all isolates harbored *aer*A and *alt* virulent genes with specific amplicons size of 301 and 442 bp, respectively (Figure 4A,B). In contrast, none of the recovered isolates was positive for the *ahp* gene (Figure 4C).

### 3.5. Restriction Fragment Length Polymorphisms (RFLP) of A. hydrophila Complex 

**Figure 5:**
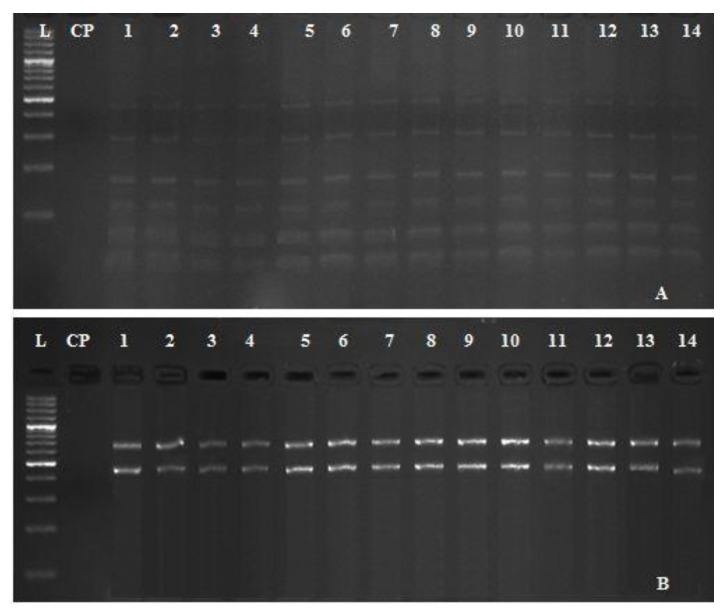
Electrophoretic patterns showing the restriction fragment length polymorphism (RFLP) analysis of *A. hydrophila* complex isolates following digestion of amplified *gyr*B gene using ECORII (**A**) and Eco31I (**B**) restriction enzymes. Lane marked L refers to 100 bp DNA ladder. CN is a negative control (DNA free template). Lanes 1–14, fragment patterns of *A. hydrophila* isolated from seven different tilapia farms (two representative isolates for each farm) located at El-Sharkia Governorate, Egypt.

The genetic diversity between 14 different isolates of *A. hydrophila* complex (two isolates per each farm), collected from seven different tilapia farms located in El-Sharkia Governorate, Egypt was investigated using two sets of restriction enzymes (ECORII and Eco31I). The results revealed that the real digestion of the *gyr*B gene gave variable banding profiles (six bands for ECORII and only two bands for Eco31I). All retrieved strains have no genetic diversity and showed dark staircase patterns of bands with identical profiles (Figure 5A,B).

### 3.6. Antimicrobial Susceptibility Testing 

The evaluated strains showed different sensitivity patterns against commercial antimicrobial agents (Table 6). Statistically, the isolated strains showed a significant difference in their susceptibility to the tested antimicrobial agents (*P <* 0.0001). 

The retrieved isolates were completely sensitive to ciprofloxacin (100%), virtually sensitive to chloramphenicol (90.10%) and trimethoprim-sulphamethoxazole (83.60%), and highly resistant to tetracycline and nalidixic acid (90.10%). Amoxicillin did not present any bactericidal activity against all evaluated strains with a resistance pattern of 100%. 

### 3.7. Pathogenicity (Challenge Trial)

**Figure 6:**
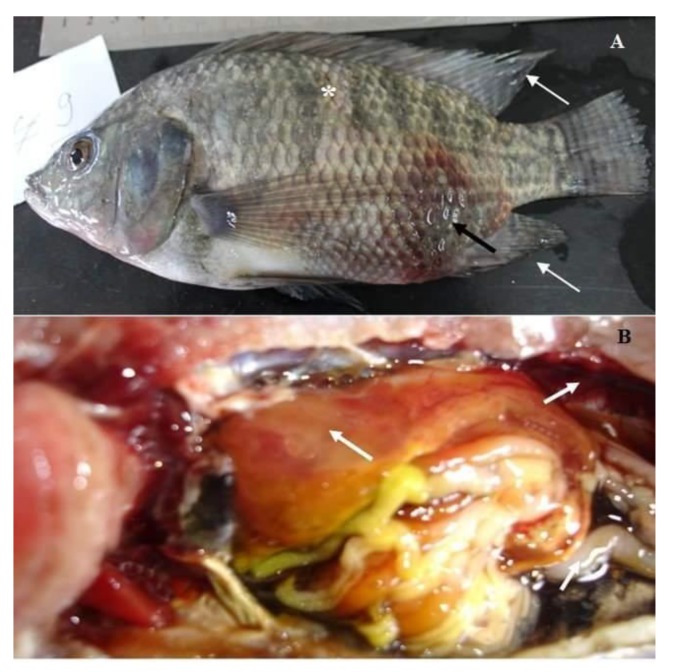
Experimentally infected *O. niloticus* with *Aeromonas hydrophila* complex showing grayish irregular ulcers on the flank region surrounded by area of erythema (black arrow), scales detachment (*), and mild fin erosions (white arrows) (**A**), friable liver, congested kidney, and serous fluid in the peritoneal cavity (**B**).

The morbidity and mortality rates of all groups experimentally challenged with *A. hydrophila* complex were evaluated for 14 days post-challenge. Fish inoculated with sterile saline (negative control) did not show any mortalities or abnormal lesions, while those subjected to 0.2 mL of *A. hydrophila* complex at a concentration of 3 × 10^8^ CFU mL^−1^ displayed high mortality (76.67%) and almost developed the same clinical signs and postmortem lesions found in naturally infected fish. Clinically infected fish showed grayish wide ulcers on the flank region surrounded by an area of erythema, dropping of the scales, slight abdominal distension, and mild fin erosions (Figure 6A). Internally, the challenged fish displayed friable liver, congested kidney, and accumulation of abdominal serous fluid (Figure 6B). Bacteria were successfully recovered from skin lesions and internal organs of freshly dead and moribund fish, and the results from the biochemical and molecular analysis proved that all isolates belong to *A. hydrophila* complex. 

## 4. Discussion

Aquaculture is the main sustainable resource of fish that can satisfy the increasing need for animal protein [33]. *Oreochromis niloticus* is the main cultured fish species in Egypt. Bacterial fish diseases are considered the main obstacles that prevent aquaculture improvement and sustainability, and resulting in high mortalities with remarkable economic losses [34]. In the present study, the clinical findings during the acute infection with *Aeromonas hydrophila* complex revealed darkness of the skin, detachment of the scales, and large irregular hemorrhages on the external body surface, while in the advanced stages, they showed hemorrhagic ulcerative lesions, fin erosions, inflamed vent, exophthalmia, and abdominal distension. Our results were nearly similar to those described in *Oreochromis niloticus* and channel catfish (*Ictalurus punctatus*) experimentally challenged with *A. hydrophila* [35,36]. Additionally, the postmortem findings revealed enlarged liver, congested spleen and kidney, sero-hemorrhagic fluids in the abdominal cavity, and friable gills, consistent with the findings of Ibrahem, et al. [37] who observed focal necrotic lesions in the liver, spleen, and kidney as well as serosanguinous fluids in the abdominal cavity of infected fish with *A. hydrophila*. The signs are attributable to bacterial extracellular proteins including, aerolysin ‘hemolytic and cytolytic’, nuclease, elastase and chitinase that induce progressive and degenerative changes in the blood vessel endothelial cells and parenchymatous organs [38].

Concerning the phenotypic characteristics of *A. hydrophila* complex as shown in Table 3, our findings emphasized no ambivalence in both morphological and biochemical characteristics and proved a magnificent harmony among various strains. The results are in accordance with those reported by HäUnninen [39] and Abbott, et al. [40]. The ability of different isolates to react positively with different biochemical tests reflects their virulence and demonstrates their ability to induce the disease. Certain biochemical reactions such as Voges Proskauer, production of acid from arabinose and sucrose, and the lysine decarboxylase test LDC test have been correlated with the virulence of *A. hydrophila* species [40]. Regarding the bacteriological examination of the diseased fish, 33 out of 250 *O. niloticus* were positive to *A. hydrophila* complex (13.2%) as described in Table 4. Furthermore, 61 isolates of *A. hydrophila* complex were retrieved from seven examined farms, while only three farms were found to be free of infection. The highest prevalence was recorded in the liver (54.10%) as shown in (Table 5). Similar findings were reported by El-Bahar, Ali, Aboyadak, Khalil and Ibrahim [32]. Indeed, there are several predisposing factors that progress the disease occurrence and increase its prevalence including; stress resulted from overcrowding of intensive systems, bad handling of fish, bad hygienic conditions, low water and bad management, insufficient oxygen, unsuitable pH and temperature [41]. 

In the present study, the results of the antibiogram assay revealed that all the tested isolates were completely sensitive to ciprofloxacin (100%), virtually sensitive to chloramphenicol (90.10%), and were highly resistant to tetracycline and nalidixic acid (90.10%). In addition, amoxicillin did not exhibit any bactericidal activity against the tested strains (Table 6). These results are nearly agreed with those obtained by Goñi-Urriza, et al. [42] and Kusdarwati, et al. [43]. The antibiotic resistance has a public health concern; it mainly results from the improper intensive use of antibiotics. In addition, the presence of antibiotic resistance genes in various movable components such as integrons and R- Plasmids is responsible for the occurrence of multidrug resistance. The resistance to penicillin is common in *A. hydrophila* complex and mainly attributed to β -lactamase production that encoded in their chromosomes [44]. *A. hydrophia* complex is commonly sensitive to quinolones “ciprofloxacin” and the resistance to such antimicrobial agents is rare which occurred due to the mutation in the *gyr*A gene [45]. Using the conventional methods for phenotypic characterization of *A. hydrophila* to the species level showed great obstacles due to its phenotypic complexity and interspecies homogeneity, as well as the prolonged-time [14]. 

Concerning the molecular typing of *A. hydrophila* complex, all the tested strains were positive for *gyr*B gene, a conserved gene of *A. hydrophila* complex with a specific amplicon size 1100 bp (Figure 3), these findings are agreed with those obtained by Yanez, Catalán, Apraiz, Figueras and Martinez-Murcia [28] and Tacão, et al. [46]. In addition, all the recovered strains harbored *aer*A and *alt* virulent genes with specific amplicons size of 301 and 442 bp, respectively (Figure 4A,B). In contrast, none of the isolated strains have harbored the *ahp* gene (Figure 4C), These results disagree with those obtained by Li et al. [29] who reported the presence of 3 virulence genes (*aer*A, *alt*, and *ahp*) in 62.7% of the tested strains. Until now the phenotypic identification of *A. hydrophila* using the conventional methods is not efficient for the differentiation of the subspecies or detection of the pathotypes. PCR plays a vital role in screening and insuring the virulence of bacterial pathogens. The pathogenicity of *A. hydrophila* complex could be investigated genetically by the determination of virulence determinant genes. The virulent *A. hydrophilla* strains always harboring two or more virulence genes including; (*aer*A, *alt*, *hly*A and *ahp*) genes [47,48].

Indeed, several studies related to *A. hydrophila* revealed variability and high genetic diversity among different strains according to their ecological distribution [49]. Therefore, the RFLP-PCR technique was performed to evaluate the genetic variability or homogeneity among representative 14 isolates of *A. hydrophila*, retrieved from seven tilapia farms in different geographical areas. RFLP analysis is an extremely useful and simplified technology that distinguishes between the most relevant and antigenically related organisms including bacteria [23]. All retrieved strains have no genetic diversity and showed dark staircase patterns of bands with identical profiles (Figure 5). The genetic stability among different strains may attribute to the short distance between the sampling points and reflect their physiological adaptation to the environments in which they exist [50].

Regarding the pathogenicity trial, fish challenged with *A. hydrophila* complex at a concentration of 3 × 10^8^ CFU mL^-1^ presented high a mortality rate (76.67%) and showed the identical signs of hemorrhagic septicemia found in the natural infected one. Thus, could be related to the fatal and pestilent effect of bacterial toxins and extracellular enzymes, which subsequently promote tissue damage and cell necrosis [51]. Most of the infected fish showed grayish wide ulcers, abdominal distension, fin erosions, friable liver, and congested kidney (Figure 6A,B), in agreement with [52]. 

## 5. Conclusions

The combination of phenotypic and genotypic analysis is a valuable epidemiological tool used in the characterization of virulent *A. hydrophila* complex. PCR is needed to confirm the identification and the pathogenicity of *A. hydrophila* complex. The most common virulence genes associated with virulent *A. hydrophila* complex are *aer*A and *alt* genes. RFLP technique is substantial for monitoring the biodiversity in the isolated *A. hydrophia* strains. The routine application of antimicrobial susceptibility testing is indispensable to select the antibiotic of choice due to the emerging multidrug resistance. Ciprofloxacin is the most effective antimicrobial agent that should be used in the treatment of Motile Aeromonas Septicemia.

## Figures and Tables

**Table 1:** List of oligonucleotide sequences and their PCR conditions used in the current study.

Target Gene	Primers Sequences 5′–3′	Amplicon Size (bp)	PCR Conditions					Reference
			No of Cycles	Denaturation	Annealing	Extension	Final Extension	
***gyr*** **B**	F: TCCGGCGGTCTGCACGGCGT R: TTGTCCGGGTTGTACTCGTC	1100	35	94 °C for 30 s	55 °C for 30 s	72 °C for 1 min	72 °C for 10 min	[28]
***aer*A**	F: AACCGAACTCTCCAT R: CGCCTTGTCCTTGTA	301	30		54 °C for 30 s			[29]
***alt***	F: TGACCCAGTCCTGG R: GGTGATCGATCACC	442	30		60 °C for 30 s			
***ahp***	F: ATTGGATCCCTGCCTA R: GCTAAGCTTGCATCCG	911	30		56 °C for 30 s			

**Table 2:** List of digestive enzymes used for restriction fragment length polymorphism (RFLP) analysis and their acronyms, sequences, frequencies and cutting positions.

Enzymes	Acronym	Sequence	Site Length	Frequency	Cut Positions
Digestive enzyme I	Eco31I	GGTCTC	6	1	690
Digestive enzyme II	EcorII	CCWGG	5	5	104, 221, 506, 983, 1055

**Table 3:** Phenotypic and biochemical characteristics of *A. hydrophila* complex isolates, retrieved from naturally infected Nile tilapia (*O. niloticus*).

Test	*A. hydrophila*
Gram stain	Gram-negative
Motility	Motile
Cell shape	Straight rods
Oxidase	+
Catalase	+
O/F	fermentative
H_2_S	−
Indole	+
Methyl red test	−
Voges Proskauer reaction	+
Vibrostatic agent or Novobiocin disc	R
Casein, Gelatin, Starch	+
Urea	−
Esculin hydrolysis Growth on	+
0–3% (*w*/*v*) sodium chloride	+
5% (*w*/*v*) sodium chloride	−
Production of acid and/or gas	
Glucose	+
Fructose	+
Salicin	+
Arabinose	V
Sorbitol	−
Sucrose	+
Mannitol	+
Inositol	+
Raffinose	−
Maltose	+
Trehalose	+

R, resistant; V, variable; +, positive; −, negative.

**Table 4:** Prevalence and distribution patterns of *A. hydrophila* complex, isolated from different farms of Nile tilapia (*O. niloticus*).

Farm No	Locality	No of Fish	Prevalence of. *A. hydrophila* Complex among Naturally Infected Fish		Chi-Square Value and Significance	
			No	%		
**1**	Abbassa	25	4	16	8.93 NS *P* > 0.05	1.7107 NS *P* > 0.05
**2**		25	6	24		
**3**		25	3	12		
**4**		25	7	28		
**5**		25	-	-		
		125	20			
**6**	El-Hossania	25	4	16	11.6758 * P < 0.05	
**7**		25	3	12		
**8**		25	6	24		
**9**		25	-	-		
**10**		25	-	-		
**Total**		250	33	13.2		

NS, non-significant. * significant difference.

**Table 5:** Prevalence of *A. hydrophila* complex in different internal organs of naturally infected Nile tilapia (*O. niloticus*).

Fish Species	Total No. of Isolates	Prevalence of Isolates in Different Organs					
		Liver		Kidney		Spleen	
		No	%	No	%	No	%
***Oreochromis niloticus***	61	33	54.1	16	26.3	12	19.6
**Chi-square value and significance**		18.3443 * *P* < 0.0001					

* significant difference.

**Table 6:** Antibiotic susceptibility of *A. hydrophila* complex isolated from naturally infected Nile tilapia (*O. niloticus*).

Antimicrobial Agents	Concentration µg	Susceptibility Patterns					
		Sensitive		Intermediate		Resistant	
		No	%	No	%	No	%
**Chloramphenicol**	30	55	90.1	6	9.9	0	0
**Trimethoprim/Sulphamethoxazol**	1.25/23.75	51	83.6	4	6.5	6	9.9
**Amoxicillin**	25	0	0	0	0	61	100
**Streptomycin**	10	11	18	13	21.3	37	60.7
**Erythromycin**	15	0	0	37	60.7	24	39.3
**Tetracycline**	30	0	0	6	9.9	55	90.1
**Ciprofloxacin**	5	61	100	0	0	0	0
**Nalidixic acid**	30	0	0	6	9.9	55	90.1
**Chi-square value and significance**		389.65 * P < 0.0001		132.17 * P < 0.0001		306.51 * P < 0.0001	

* significant difference.
